# Acute Toxicity, Neurotoxic, Immunotoxic, and Behavioral Effects of Deltamethrin and Sulfamethoxazole in Adult Zebrafish: Insights into Chemical Interactions and Environmental Implications

**DOI:** 10.3390/toxics13020128

**Published:** 2025-02-10

**Authors:** Yueyue Liu, Fengyu Liu, Chen Wang

**Affiliations:** 1 College of Water Science, Beijing Normal University, Beijing 100875, China; liuyueyue0103@mail.bnu.edu.cn; 2 State Key Laboratory of Environmental Criteria and Risk Assessment, Chinese Research Academy of Environmental Sciences, Beijing 100012, China; 3 State Environment Protection Key Laboratory of Environmental Monitoring Quality Control, China National Environmental Monitoring Center, Beijing 100012, China; liufengyu@cnemc.cn

**Keywords:** pyrethroids, antibiotic, immunotoxicity, neurotoxicity, zebrafish

## Abstract

The increasing presence of antimicrobial agents and pesticides in aquatic environments raises concerns about their potential impacts on non-target organisms. Among these chemicals, deltamethrin (DM), a widely used pesticide, and sulfamethoxazole (SMX), an antimicrobial commonly detected in water bodies, pose significant ecological risks. This study investigates the acute toxicity, neurotoxic effects, oxidative stress responses, immune-related gene expression, and feeding behavior of adult zebrafish exposed to DM and SMX. The 96 h LC_50_ for DM was 4.84 µg/L, indicating significant acute toxicity, while the LC_50_ for the DM + SMX mixture was 11.32 µg/L, suggesting that SMX may mitigate the toxicity of DM. Neurotransmitter alterations, including reduced levels of γ-aminobutyric acid (γ-GABA), serotonin (5-HT), and acetylcholinesterase (AChE), were observed, with the combination of DM and SMX showing partial restoration of AChE activity. Oxidative stress markers revealed significant changes in antioxidant enzyme activities, with DM exposure increasing superoxide dismutase (SOD) and glutathione-S-transferase (GST) activities, while decreasing catalase (CAT) and glutathione peroxidase (GPX) activities. Immune-related gene expression demonstrated suppressed IgM, IgD, and IgZ levels, along with altered inflammatory responses, with both DM and DM + SMX exposure inducing pro-inflammatory cytokines. Finally, feeding behavior was significantly impaired in the DM group at the 3 min mark, while the DM + SMX group showed partial mitigation of this effect. These findings highlight the neurotoxic, immunotoxic, and behavioral effects of DM and SMX, and underscore the potential for chemical interactions to modulate toxicity in aquatic organisms.

## 1. Introduction

Deltamethrin (DM), a widely used pyrethroid insecticide, has raised significant concerns due to its neurotoxic effects on aquatic organisms, including fish [1,2]. Pyrethroids like DM exert their toxicity primarily by disrupting sodium ion channels in the nervous system, which leads to excessive neural stimulation and, ultimately, mortality in aquatic species [3]. As a commonly applied pesticide, DM is frequently detected in aquatic ecosystems, such as rivers, lakes, and streams, particularly those affected by runoff from agricultural fields, where it becomes a major environmental contaminant [4,5]. The concentrations of DM are reported to range from ng/L to µg/L [6,7,8,9], which are high enough to pose a risk to aquatic organisms, especially in the case of chronic exposure or during periods of heavy runoff. Sulfamethoxazole (SMX) is a widely used synthetic antimicrobial agent belonging to the sulfonamide class of antibiotics [10]. It is commonly widely used in both human medicine and aquaculture to treat a variety of bacterial infections [11,12]. SMX is also frequently detected in aquatic environments due to its widespread use and limited degradation in natural water bodies [11,13]. As another pervasive contaminant in water systems, SMX poses a potential threat to aquatic organisms, as it can accumulate in water and affect the health of aquatic species, including fish [12,14].

The environmental persistence of SMX, combined with its frequent co-occurrence with other pollutants, such as pesticides, raises concerns about the potential synergistic or antagonistic effects of chemical mixtures on aquatic organisms. Recent studies indicated that mixtures of environmental contaminants can alter the toxicity of individual chemicals through various mechanisms, including metabolic interactions and changes in bioavailability [15,16,17]. The toxicological effects of individual chemicals have been extensively studied. However, there is limited research on the combined toxicity of pesticides and antibiotics, particularly concerning neurotoxicity, oxidative stress, and immune dysfunction in aquatic species. Given the frequent detection of these chemicals together in aquatic environments, it is essential to explore how their combination impacts the health of aquatic organisms.

Zebrafish (Danio rerio) have emerged as a prominent model organism for toxicological studies due to their well-documented sensitivity to environmental contaminants and their physiological similarity to other vertebrates, including humans [18,19]. This study investigates the acute toxicity of DM, both individually and in combination with SMX, in adult male zebrafish to explore the potential neurotoxic, immunotoxic, and behavioral effects of these chemicals. Altered feeding behavior often signals disruptions in neurological function, energy metabolism, or general health [20]. Given that pyrethroids like DM are known to affect neural pathways, we hypothesized that exposure to DM may impair zebrafish feeding behavior. Furthermore, we aimed to evaluate whether the presence of SMX modulates these effects. In addition to behavioral changes, we also investigated the effects of these chemicals on neurotransmitter levels, immune-related gene expression, and oxidative stress markers in the brain. These biomarkers are crucial for understanding the molecular mechanisms underlying toxicity, as they provide insights into the disruption of neurochemical signaling, immune function, and cellular stress responses, which may have long-term implications for fish populations and aquatic ecosystems. The neurotransmitters γ-aminobutyric acid (GABA), serotonin (5-HT), and acetylcholinesterase (AChE) are key indicators of neurotoxicity in zebrafish [21], with alterations in their levels reflecting disturbances in neural signaling [22,23]. Moreover, oxidative stress and immune dysfunction, which are often induced by chemical exposure, can contribute to long-term neurotoxic effects [24,25,26].

Overall, this study seeks to address this gap by investigating the acute toxicity, neurotoxic effects, and immune-related impacts of DM, both individually and in combination with SMX, in adult zebrafish. Specifically, we aim to examine alterations in neurotransmitter alterations, oxidative stress markers, and immune function, all of which are key indicators of chemical-induced neurotoxicity and systemic disruption. By investigating these endpoints, we aim to better understand the potential risks posed by the co-occurrence of pesticides and antibiotics in aquatic environments and the implications for the health of aquatic organisms and inform future risk assessment efforts for aquatic ecosystems.

## 2. Materials and Methods

### 2.1. Chemicals and Reagents

Deltamethrin (≥98% purity), sulfamethoxazole (99% purity), and dimethyl sulfoxide (DMSO, chromatographic grade) were purchased from Sigma-Aldrich (St. Louis, MO, USA). Phosphate-buffered saline (PBS) was obtained from Solarbio (Beijing, China). MS-222 (tricaine methanesulfonate), at a concentration of 100 mg/L, was purchased from Sigma-Aldrich (St. Louis, MO, USA). The DM and SMX were dissolved in DMSO to obtain a stock solution and then diluted with water to achieve the desired test concentrations.

### 2.2. Zebrafish Maintenance and Exposure Design

Wild-type AB adult zebrafish (approximately 6 months old) were obtained from the State Key Laboratory of the Chinese Academy of Environmental Sciences. The fish were maintained in a flow-through system at 27 ± 1 °C, 50 ± 10% relative humidity, and a 14 h:10 h light:dark cycle. Continuous aeration in water was employed to maintain dissolved oxygen levels above 7.72 mg/L, while conductivity was kept at 480 μS/cm, with minor fluctuations in these values possible. The fish were acclimatized for 14 days prior to the start of the experiment, during which they were fed freshly hatched brine shrimp (*Artemia nauplii*) twice daily. All experiments were conducted in accordance with the Guidelines for the Keeping and Use of Laboratory Animals of the Chinese Academy of Environmental Sciences. For the acute toxicity test, we conducted the experiment based on the OECD Test Guideline No. 203 for Fish, Acute Toxicity Testing. To avoid potential reproductive or hormonal differences that could influence behavior and neurochemical responses, only male zebrafish were used to assess the toxic effects of DM and SMX in this study. Adult male zebrafish (10 fish per parallel, 6 parallels per concentration group) were exposed to different concentrations of DM (0, 1.0, 2.0, 4.0, 8.0, and 16.0 μg/L) in a clear glass beaker containing 2 L of water for 96 h. Additional groups were exposed to the same concentrations of DM in combination with SMX (30 μg/L) for the same duration. The concentrations of SMX were selected based on environmentally relevant levels [10]. The DM concentrations were chosen based on prior toxicity studies in zebrafish, which demonstrated toxic effects at these concentrations and provided a foundation for evaluating potential ecological risks [6,27].

Each concentration was tested in six replicates to ensure statistical reliability. Control groups were exposed to DMSO at the same concentration as in the experimental groups (0.001% DMSO). Fish were observed for signs of acute toxicity, including mortality, abnormal swimming behavior, and other physical changes (e.g., coloration, fin damage, or erratic movement). At the end of the exposure period (96 h), fish were euthanized using an overdose of MS-222 and thoroughly rinsed and sterilized with PBS, and the following endpoints (mortality, behavioral changes, and immunotoxicity) were assessed. Based on the LC_50_ values, the concentration closest to the LC_50_ was selected for further analysis (4.0 μg/L). This concentration was chosen because it represents a sublethal concentration that ensures sufficient survival rates and it is also suitable for evaluating potential sublethal effects. The control group (0 μg/L) was used to compare and evaluate the baseline endpoints under normal conditions.

### 2.3. Feeding Behavior Assessment

Food intake was analyzed to assess feeding behavior based on previous studies [20,28]. After 96 h of exposure, a total of 15 zebrafish from each treatment group (2 or 3 in each tank) were randomly collected and placed into three 2 L glass tanks, with five zebrafish per tank and 1 L of clean water in each tank. After a 2 h acclimation period, the zebrafish were fed 30 commercially available food pellets (average diameter = 1 mm; Yee Corp., Weifang, China). The number of pellets consumed by the zebrafish was recorded at 3, 5, and 10 min post-feeding to assess their feeding behavior. The results were expressed as the ratio of the number of pellets consumed by the zebrafish to the total number of pellets provided.

### 2.4. Assessment of Neurotransmitter and AChE Levels

The zebrafish brain was carefully dissected, ensuring that the tissue was kept on ice throughout the process to minimize enzymatic degradation. Each brain sample was collected in an ice-cold PBS solution. The brain tissue was homogenized in 10 volumes of ice-cold PBS using a tissue homogenizer or a glass homogenizer. The homogenate was then centrifuged at 4 °C for 15 min at 5000× *g* to remove debris. The supernatant was collected and stored at −80 °C for subsequent analysis.

The total protein concentration in each sample was first measured using a BCA assay, and the data were normalized. The activity of AChE was determined by the enzyme-linked immunosorbent assay with commercial kits (Nanjing Jiancheng Bioengineering Institute, Nanjing, China), following the manufacturer’s instructions. The contents of 5-HT and gamma aminobutyric acid (γ-GABA) were measured using the enzyme-linked immunosorbent assay (ELISA) (Shanghai Enzyme-Linked Biotechnology Co., Ltd., Shanghai, China) according to the manufacturer’s guidelines. The absorbance (OD value) of AChE and three neurotransmitters was measured by the double-antibody sandwich assay at a wavelength of 450 nm using a enzyme-labeled instrument (BioTek Epoch 2, Agilent Technologies, Inc., Santa Clara, CA, USA). The concentrations of AChE and the three neurotransmitters in the zebrafish samples were calculated using the linear regression equation derived from the standard curve, which was established by plotting the concentrations of the standards against their corresponding OD values. To meet the required sample size for the experiment, we collected the brains from two zebrafish per replicate. Each group included 4 biological replicates (n = 4), and enzyme activity measurements were performed in duplicate.

### 2.5. Analysis of Oxidative Stress Enzyme Activity

Firstly, zebrafish brain tissue was accurately weighed and added to 9 times the volume of saline in the ratio of weight (g):volume (mL), 1:9, of physiological saline. Mechanical homogenization was performed in an ice-water bath, followed by Eppendorf 5804R centrifugation at 2500 RCF for 10 min(Eppendorf, Hamburg, Germany) and determination of protein content in the supernatant using the kit (Nanjing Jiancheng Bioengineering Institute, Nanjing, China). The activities of superoxide dismutase (SOD), catalase (CAT), glutathione peroxidase (GPX), and glutathione S-transferase (GST) were determined using commercial kits (Nanjing Jiancheng Bioengineering Institute, Nanjing, China) following the manufacturers’ instructions. For the assessment of oxidative stress enzyme activity, each treatment group (including the control and experimental groups) consisted of 4 biological replicates (n = 4), and each replicate contained two zebrafish brain tissues. The enzyme activities were measured in duplicate to ensure reliability and consistency of the data.

### 2.6. Real-Time Quantitative Polymerase Chain Reaction (RT-qPCR) Analysis

Following the 96 h exposure, zebrafish brain tissues were collected for measuring the expression of immune genes to assess the effects of exposure on organisms. Total RNA was extracted from the brain tissues using a commercially available RNA extraction kit (Thermo Fisher Scientific, Waltham, MA, USA) according to the manufacturer’s protocol. The extracted RNA was quantified and checked for purity (A260/A280 ratio) by using a Nanodrop ND-2000 spectrophotometer (Thermo Fisher Scientific Inc., Waltham, MA, USA). RNA (1 μg) was reverse transcribed into complementary DNA (cDNA) using a high-capacity cDNA reverse transcription kit (Roche Diagnostics, Basel, Switzerland) following the manufacturer’s instructions. RT-qPCR was performed using a Fluorescent Quantitative PCR Detection System (LineGene 9600, Hangzhou Bioer Technology Co., Ltd., Hangzhou, China) with the FastStart Universal SYBR Green Master (ROX) kit (Roche). Primer sequences for target immune-related genes and housekeeping (RPL13a) genes were referenced to previous literature, and the detailed primer sequences are shown in Table S1. Primers were synthesized by Sangon Biotech (Shanghai, China). The relative expression of the genes was performed for each gene with three technical replicates and three biological replicates (n = 3) and was calculated using the 2^−ΔΔCt^ method.

### 2.7. Data Statistics and Analysis

The LC_50_ values for deltamethrin and sulfamethoxazole were calculated using Origin 2023 (OriginLab, Northampton, MA, USA). Mortality rates were expressed as percentages for each concentration, and the dose–response data were fitted to a logistic regression model using the Nonlinear Curve Fit tool. The LC_50_ value was derived from the concentration corresponding to 50% mortality based on the logistic equation. The goodness of fit for the logistic model was evaluated using the coefficient of determination (R^2^), with a value closer to 1 indicating a good fit. The confidence intervals for the LC_50_ values were calculated, and the precision of the estimates was assessed. Prior to statistical analysis, the normality of the data was evaluated using the Shapiro–Wilk test, which confirmed that the data followed a normal distribution. Therefore, a one-way ANOVA was conducted to analyze the effects on immunogenes, ingestion, neurotransmitters, and oxidative stress enzymes by using GraphPad Prism 9.0 (San Diego, CA, USA). Dunnett’s test was used to identify significant differences between the control and treatment groups. Data are presented as mean ± standard deviation. Differences between groups were considered statistically significant at *p* < 0.05 (*), and highly significant at *p* < 0.01 (**).

## 3. Results and Discussion

### 3.1. Acute Toxicity of DM and SMX in Adult Zebrafish

**Figure 1:**
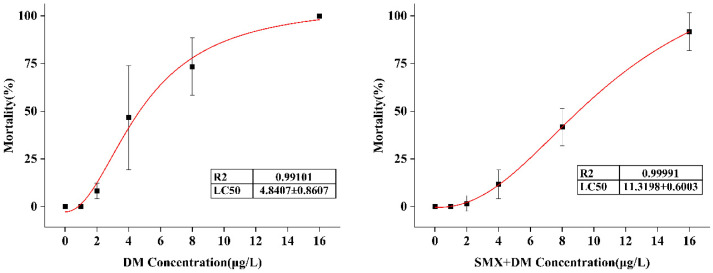
The 96 h LC_50_ results of the acute toxicity test for deltamethrin (DM) and sulfamethoxazole (SMX) on adult zebrafish. The LC_50_ values were determined by plotting the concentration of each chemical against the percentage of mortality, with data points representing the mean mortality rate from multiple replicates. The dose–response curves were fitted using logistic regression, and LC_50_ values were calculated from the concentration corresponding to 50% mortality. Statistical analysis was performed to assess significant differences between the two chemicals.

The acute toxicity of DM and SMX was evaluated by calculating the 96 h LC_50_ for adult zebrafish based on statistical mortality, as shown in Figure S1. The LC_50_ for deltamethrin was found to be 4.84 µg/L, indicating significant acute toxicity at relatively low concentrations (Figure 1). The relatively low LC_50_ observed in this study is similar to previous reports highlighting the neurotoxic potential of pyrethroids to aquatic species [29]. Interestingly, the toxicity of the deltamethrin–SMX mixture was lower than that of deltamethrin alone, with the LC_50_ of the mixture being 11.32 µg/L, which is almost twice the LC_50_ of deltamethrin alone. This result suggests a protective or mitigating effect of SMX in reducing the acute toxicity of deltamethrin at the tested concentration. SMX is widely used in aquaculture to control various bacterial diseases due to its ability to inhibit the folate metabolism pathway in bacteria [10]. SMX may alter the pharmacokinetics or bioavailability of deltamethrin, thereby reducing the toxic effects. Deltamethrin exposure in adult zebrafish has been shown to result in similar LC_50_ values as those observed in the current study [30]. These findings suggest that the presence of additional contaminants, such as sulfamethoxazole, may alter deltamethrin toxicity.

### 3.2. Neurotransmitter Alterations in the Zebrafish Brain Following Chemical Exposure

**Figure 2:**
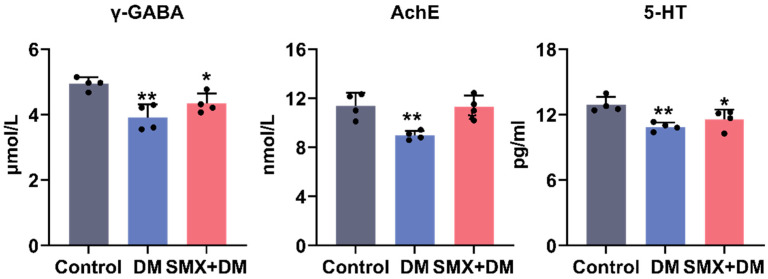
Changes in the levels of major neurotransmitters in the zebrafish brain following 96 h of exposure. Data are shown as mean ± standard deviation (n = 4). Statistical differences between control and exposed groups were assessed using one-way ANOVA, followed by Dunnett’s test. As * indicate a significant difference (*p* < 0.05) or ** indicated highly significant (*p* < 0.01) differences.

To investigate the neurotoxic effects of DM and its combination with SMX on neurotransmitter levels in adult zebrafish, we quantified γ-GABA, 5-HT, and AChE in the brain 96 h following acute exposure to 4 µg/L of DM and a combination of 4 µg/L of DM and 30 µg/L of SMX (Figure 2). The level of γ-GABA in the brain was significantly altered across the treatment groups. In the control group, the γ-GABA level was 4.94 ± 0.19 µmol/L, while in the DM group and the DM + SMX group, the levels were significantly reduced to 3.91 ± 0.39 µmol/L (*p* = 0.002) and 4.34 ± 0.30 µmol/L (*p* = 0.040), respectively. The significant decrease in γ-GABA levels following DM exposure suggests an impairment of inhibitory neurotransmission, which could be indicative of disruptions in GABAergic signaling pathways [31]. GABA is a crucial inhibitory neurotransmitter in the brain, and alterations in its levels may be associated with neurological disorders, including seizures and anxiety-like behavior [32]. The combined exposure to DM and SMX did not further exacerbate the reduction in γ-GABA levels, suggesting that while SMX may not directly affect GABAergic systems, its presence does not significantly enhance the neurotoxic effects of deltamethrin on GABAergic signaling. This finding indicates that the impact of SMX on neurotransmitter systems may be less pronounced compared to deltamethrin, and that any observed alterations in γ-GABA levels are primarily driven by deltamethrin exposure.

Similarly, the 5-HT levels were found to be lower in both the DM− and DM + SMX-exposed groups compared to the control. The 5-HT concentration was 12.91 ± 0.71 pg/L in the control group, which significantly decreased to 10.88 ± 0.40 pg/L in the DM group (*p* < 0.01) and 11.57 ± 0.90 pg/L (*p* < 0.05) in the DM + SMX group. This suggests potential disturbances in serotonergic signaling, which plays a key role in regulating mood, behavior, and neuroendocrine functions. The reduction in 5-HT, particularly in the DM group, could be linked to the neurotoxic effects of DM, which has been previously reported to affect serotonin receptors and transporters in various organisms [1,33]. The AChE activity was also significantly affected by the chemical exposures. The control group exhibited a level of 11.40 ± 1.03 nmol/L, while the DM group showed a notable decrease to 8.99 ± 0.40 nµmol/L. In the DM + SMX group, the AChE level was partially restored to 11.28 ± 0.94 nmol/L, although this was still lower than the control group. Statistical analysis confirmed a significant reduction in AChE activity in the DM group, but no significant change was observed between the DM + SMX and control groups. AChE activity is essential for the hydrolysis of acetylcholine at synapses, and its inhibition can lead to overstimulation of cholinergic receptors, with subsequent neurological effects [34]. The significant reduction in AChE activity in the DM group indicates potential cholinergic dysfunction, which may contribute to neurobehavioral alterations [27]. Interestingly, the partial restoration of AChE activity in the DM + SMX group suggests that sulfamethoxazole might modulate the impact of cyfluthrin on cholinergic systems, although the exact mechanisms remain unclear.

### 3.3. Oxidative Stress Response in the Zebrafish Brain After Chemical Exposure

**Figure 3:**
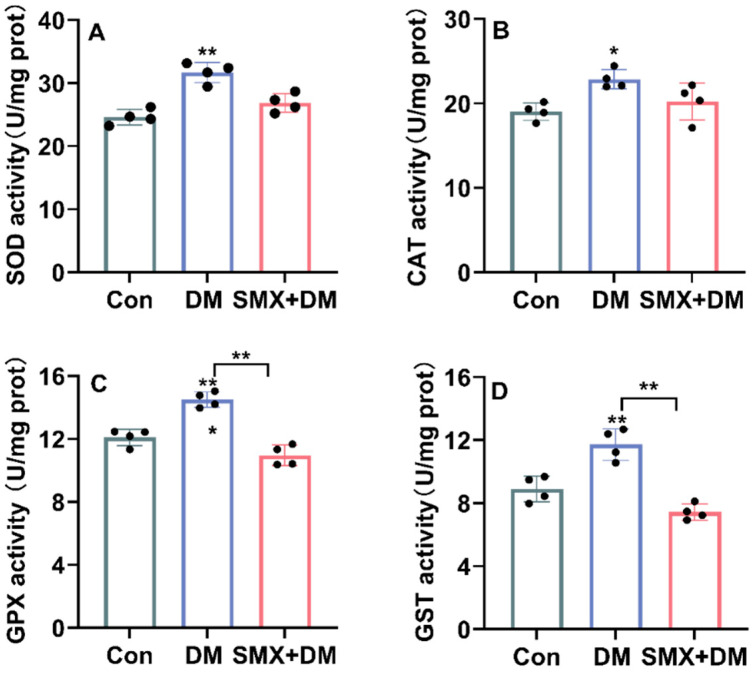
Levels of SOD (**A**), CAT (**B**), GPX (**C**) and GST (**D**) in the zebrafish brain following exposure. Data are expressed as mean ± standard deviation. Statistical analysis was performed using one-way ANOVA, followed by Dunnett’s test. Significance levels were denoted as * (*p* < 0.05) for significant differences and ** (*p* < 0.01) for highly significant differences.

Previous studies have reported oxidative stress effects in zebrafish exposed to deltamethrin (DM), although the specific responses can vary depending on exposure conditions and chemical interactions [27]. To investigate the oxidative stress response in zebrafish brains after acute exposure to 4 µg/L of DM and the combination of 4 µg/L of DM with 30 µg/L of DM + SMX for 96 h, we measured the activities of key antioxidant enzymes, including superoxide SOD, CAT, GPX, and GST (Figure 3).

The results revealed a significant alteration in the levels of these enzymes in the zebrafish brain after chemical exposure. In the control group, the SOD activity was 24.62 ± 1.24 U/mg protein, but it significantly increased to 31.68 ± 1.60 U/mg protein in the DM group (*p* < 0.01) and 26.87 ± 1.48 U/mg protein in the DM + SMX group (Figure 3). The increase in SOD activity in the DM group suggests a compensatory response to oxidative stress induced by cyfluthrin exposure. SOD is the first line of defense against reactive oxygen species (ROS) by catalyzing the conversion of superoxide anions into hydrogen peroxide (H_2_O_2_), which may indicate an attempt by the zebrafish brain to counteract oxidative damage [35,36]. However, the decrease in SOD activity in the DM + SMX group compared to the DM group suggests that the combination of cyfluthrin and sulfamethoxazole may impair this compensatory mechanism, leading to less efficient ROS neutralization. For CAT, a key enzyme involved in H_2_O_2_ detoxification [36], the control group exhibited an activity of 19.03 ± 1.04 U/mg protein. After chemical exposure, the levels were significantly increased to 22.87 ± 1.13 U/mg protein in the DM group (*p* < 0.05) and 20.21 ± 2.20 U/mg protein in the DM + SMX group. These reductions reflect a disruption in the detoxification pathway for hydrogen peroxide, a potentially harmful byproduct of cellular metabolism [37]. The observed decrease in CAT activity in both the DM and DM + SMX groups is consistent with the heightened oxidative stress due to chemical exposure, which may overwhelm the cellular antioxidant defenses. Previous studies have reported that DM exposure has been shown to induce oxidative stress in various aquatic organisms, including zebrafish, by disrupting the antioxidant defense systems [38], which supports our findings of significant changes in SOD and CAT activity in zebrafish brain tissues. For instance, a study demonstrated that exposure to deltamethrin led to a significant decrease in SOD and CAT activities in zebrafish liver, suggesting oxidative damage as a result of pesticide toxicity [2]. Similarly, a review study reported that DM exposure in zebrafish embryos resulted in increased oxidative stress markers, including lipid peroxidation, and impaired antioxidant enzyme activities, such as SOD and CAT [27], which aligns with the findings in our study showing an increase in SOD activity in the DM group and a decrease in CAT activity in both the DM and DM + SMX groups.

The GPX activity in the control group was 12.10 ± 0.52 U/mg protein. In the DM group, GPX activity was significantly reduced to 11.50 ± 0.49 U/mg protein (*p* < 0.01), and it further decreased to 10.95 ± 0.67 U/mg protein in the DM + SMX group. GPX is crucial for reducing lipid peroxides and protecting cell membranes from oxidative damage [37]. The reduction in GPX activity suggests that both cyfluthrin and its combination with sulfamethoxazole induced oxidative damage in zebrafish brain cells, compromising the organism’s ability to mitigate lipid peroxidation and oxidative injury [24,39].

GST activity, which plays an important role in detoxification by conjugating reactive electrophiles to glutathione [40], was 8.90 ± 0.82 U/mg protein in the control group. In the DM group, the activity significantly increased to 11.72 ± 0.99 U/mg protein (*p* < 0.01), while in the DM + SMX group, it decreased to 7.44 ± 0.51 U/mg protein. The elevated GST activity in the DM group suggests an enhanced detoxification response to cyfluthrin exposure, possibly due to the increased production of reactive intermediates. However, the decrease in GST activity in the DM + SMX group may indicate an additive or synergistic toxic effect of the two chemicals, which could impair the zebrafish’s ability to detoxify harmful compounds, leading to an exacerbation of oxidative stress.

### 3.4. Changes in Immune-Related Gene Expression in the Zebrafish Brain

**Figure 4:**
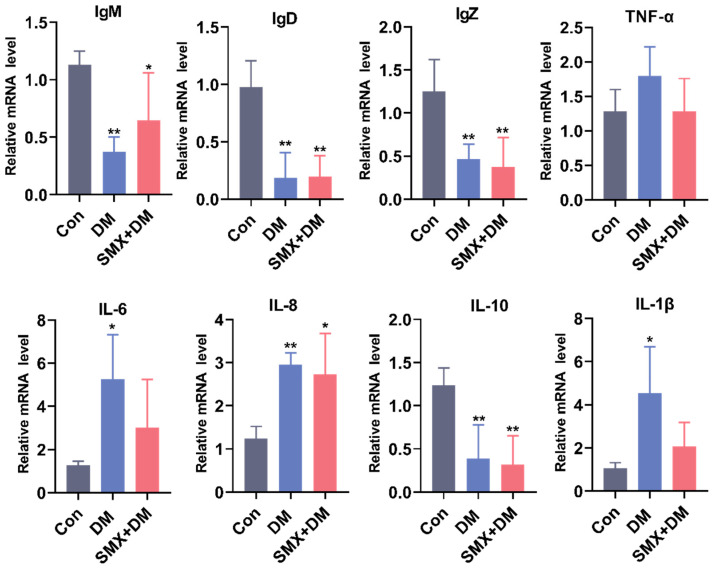
Alterations in the expression of immune-related genes in the zebrafish brain following 96 h of exposure. Gene expression was quantified using quantitative PCR (qPCR) and normalized to the housekeeping gene. Data are presented as fold changes relative to the control group. Statistical significance was determined by one-way ANOVA, followed by Dunnett’s test. Statistical significance was indicated by asterisks: * *p* < 0.05 and ** *p* < 0.01.

We investigated changes in immune-related gene expression in the zebrafish brain following exposure to DM and SMX. Specifically, we assessed the expression of immunoglobulin genes (IgM, IgD, and IgZ) and inflammatory cytokines to understand the immune response alterations induced by these chemicals (Figure 4).

The expression of IgM, IgD, and IgZ in the zebrafish brain was significantly reduced in both the DM and DM + SMX treatment groups compared to the control group. Specifically, IgM expression decreased from 1.13 ± 0.11-fold in the control group to 0.37 ± 0.13-fold (*p* < 0.01) in the DM group and 0.65 ± 0.41-fold (*p* < 0.05) in the DM + SMX group (Figure 4). Similarly, IgD expression was significantly downregulated in the treatment groups, dropping to 0.19 ± 0.22-fold (*p* < 0.01) in the DM group and 0.20 ± 0.18-fold (*p* < 0.01) in the DM + SMX group, compared to 0.98 ± 0.23-fold in the control group. For IgZ, the control group showed an expression of 1.25 ± 0.36-fold, while both the DM and DM + SMX groups exhibited significant reductions to 0.46 ± 0.17-fold (*p* < 0.01) and 0.37 ± 0.34-fold (*p* < 0.01), respectively. These findings suggest that exposure to DM and SMX suppresses immune-related pathways, particularly those involving immunoglobulins that are crucial for both systemic and mucosal immunity in zebrafish [41], potentially impairing their ability to produce antibodies in response to pathogens.

We also assessed the expression of anti-inflammatory cytokine IL-10. The control group exhibited an expression of 1.24 ± 0.20-fold, whereas the DM and DM + SMX groups had significantly lower expression levels of 0.39 ± 0.39-fold (*p* < 0.01) and 0.32 ± 0.33-fold (*p* < 0.01), respectively. IL-10 is known to regulate inflammatory responses, and its suppression may indicate an impaired ability of the zebrafish to resolve inflammation [42], potentially leading to prolonged immune activation and tissue damage [43,44]. In contrast, IL-8, a pro-inflammatory cytokine, showed an upregulation in both treated groups. The control group exhibited a baseline expression of 1.24 ± 0.28-fold, while the DM and DM + SMX groups had significantly higher levels of 2.95 ± 0.27-fold (*p* < 0.01) and 2.73 ± 0.95-fold (*p* < 0.05), respectively. This suggests that both deltamethrin and sulfamethoxazole induce a pro-inflammatory response in the zebrafish brain, likely triggering the activation of innate immune pathways. IL-8 plays a role in neutrophil recruitment and the overall inflammatory cascade, and its elevation points to an exacerbated inflammatory environment in the brain following chemical exposure [45]. The expression of pro-inflammatory cytokines IL-6 and IL-1β was significantly elevated only in the DM group, with no significant change observed in the DM + SMX group. IL-6 and IL-1β are key players in the initiation of acute inflammation and are known to activate downstream immune signaling pathways [44]. The observed increase in these markers in the DM group suggests that deltamethrin specifically induces an inflammatory response, which may contribute to neuroinflammation and potential neurotoxic effects. Interestingly, the expression of TNF-α did not show significant changes in either the DM or DM + SMX groups compared to the control. Overall, these results highlighted the potential immunotoxic effects of these chemicals, which could impair immune function and contribute to neuroinflammation in aquatic organisms.

### 3.5. Effects of Deltamethrin and Sulfamethoxazole on Feeding Behavior of Zebrafish

**Figure 5:**
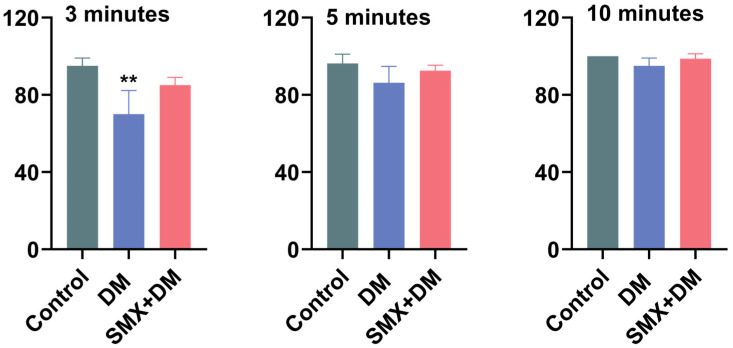
Effects of DM and SMX exposure on the feeding behavior of zebrafish. Statistical differences between the control and exposed groups were assessed using one-way ANOVA, followed by Dunnett’s multiple comparison test. ** for *p* < 0.01, indicating highly significant differences.

The feeding behavior of zebrafish was assessed by recording the number of pellets consumed at 3, 5, and 10 min after feeding, with results expressed as the ratio of consumed pellets to the total provided (Figure 5). At 3 min, the control group consumed 95.00 ± 4.08% of the pellets, while the DM group showed a significantly lower feeding rate of 70.00 ± 12.25% (*p* < 0.01), which suggested that DM exposure negatively impacts the immediate feeding behavior of zebrafish. However, the DM + SMX group did not significantly alter feeding behavior (85.00 ± 4.08%), indicating a potential mitigating effect of sulfamethoxazole on the feeding impairments caused by deltamethrin. Although feeding rates in the DM and DM + SMX groups slightly decreased at the 5 and 10 min time points compared to the control, no significant differences were observed at these time intervals. The impact on feeding behavior seemed to be short-lived, with no significant differences at later time points.

## 4. Conclusions

The results of this study demonstrated that DM induced significant acute toxicity, neurotoxic effects, and oxidative stress in zebrafish, while SMX appeared to mitigate some of these effects, particularly in terms of feeding behavior and AChE activity. The combination of DM and SMX did not exacerbate the neurotoxic effects, but partially restored AChE activity. Additionally, oxidative stress responses and immune system alterations suggested that these chemicals can impair the zebrafish’s ability to cope with environmental stressors. The observed decrease in immune-related gene expression and increase in pro-inflammatory cytokines indicated potential immunotoxicity, which may contribute to neuroinflammation. These findings underscore the importance of considering chemical interactions in environmental risk assessments and highlight the need for further research to explore the underlying mechanisms of toxicity and their long-term impacts on aquatic ecosystems.

## Data Availability

The datasets used and/or analyzed during the current study are available from the corresponding author upon reasonable request.

## Supplementary Materials

The following supporting information can be downloaded at: https://www.mdpi.com/article/10.3390/toxics13020128/s1. Figure S1: Mortality of zebrafish after 96 h of acute exposure to different concentrations of DM and SMX. Table S1: Primers used for real-time quantative PCR assays [28,46].
