# Supplementary material for: Acute Toxicity, Neurotoxic, Immunotoxic, and Behavioral Effects of Deltamethrin and Sulfamethoxazole in Adult Zebrafish: Insights into Chemical Interactions and Environmental Implications
Source: Toxics. 2025 Feb 10;13(2):128. doi: 10.3390/toxics13020128 (PMC11860506; doi:10.3390/toxics13020128)
Supplement: Supplementary file 1 [file toxics-13-00128-s001.zip › toxics-3440554-supplementary.pdf]

Article

Acute Toxicity, Neurotoxic, Immunotoxic, and Behavioral Effects of Deltamethrin and Sulfamethoxazole in Adult Zebrafish: Insights into Chemical Interactions and Environmental Implications

Yueyue Liu[1,2], Fengyu Liu[3], Chen Wang[2,*]

[1] College of Water Science, Beijing Normal University, Beijing, 100875, China.
[2] State key laboratory of Environmental Criteria and Risk Assessment, Chinese Research Academy of Environmental Sciences, Beijing,100012, China.
[3] State Environment Protection Key laboratory of Environmental Monitoring Quality Control, China National Environmental Monitoring Center, Beijing 100012, China.
* Correspondence: wangchen@craes.org.cn (C.W.)

This PDF file includes:

Figures S1
Tables S1
Supplementary References

Table S1. Primers used for real-time quantative PCR assays[1, 2]

Genes	Accession No.	Primer sequences
RpL13a	NM_212784.1	Forward：TGGTGAGGTGTGAGGGTATCAAC
		Reverse：AATTTGCGTGTGGGTTTCAGAC
IgM	AY643751.1	Forward：GAAGCCTCCAATTCTGTTGG
		Reverse：CCGGGCTAAACACATGAAG
IgD	XM_021474792.1	Forward：GACACATTAGCCCATCAGCA-
		Reverse：CTGGAGAGCAGCAAAAGGAT
IgZ	AY643750.1	Forward：GAACCAAACTCAGGGTTGGA
		Reverse：CACCCAGCATTCTACAGCAA
IL6	NM_001261449.1	Forward：TCCTGGTGAACGACATCAAA
		Reverse：TCATCACGCTGGAGAAGTTG
IL 10	NM_001020785.2	Forward：CAGTCCCTATGGATGTCACG
		Reverse：CCGCTTGAGTTCCTGAAA
IL 1β	NM_212844.2	Forward：CATTTGCAGGCCGTCACA
		Reverse：GGACATGCTGAAGCGCACTT
TNFα	NM_212859.2	Forward：CCATGCAGTGATGCGCTTT
		Reverse：TTGAGCGGATTGCACTGAAA
IL8	XM_001342570.8	Forward：GTCGCTGCATTGAAACAGAA
		Reverse：CTTAACCCATGGAGCAGAGG

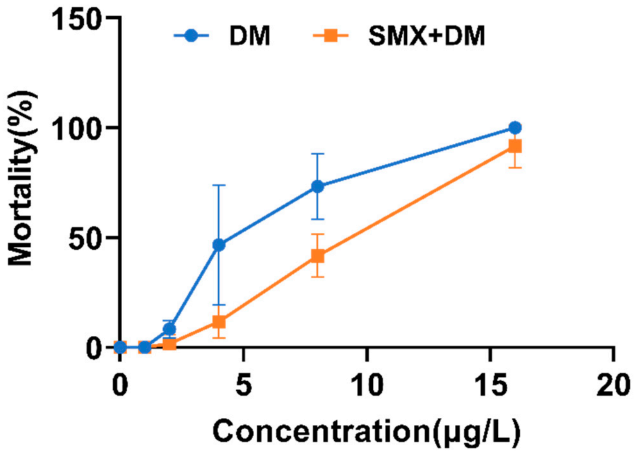

Figure.S1. Mortality of zebrafish after 96 hours of acute exposure to different concentrations of DM and SMX (n = 6).

References

1. Medina-Gali, R.M.; Ortega-Villaizan, M.D.M.; Mercado, L.; Novoa, B.; Coll, J.; Perez, L. Beta-glucan enhances the response to SVCV infection in zebrafish. *Dev. Comp. Immunol.* **2018**, *84*, 307–314.
2. Liu, Y.; Lin, S.; Wang, C.; Li, T.; Zheng, G.; Sun, W.; An, L.; Bai, Y.; Wu, F. Sex-Specific Effects of Environmental Exposure to the Antimicrobial Agents Benzalkonium Chloride and Triclosan on the Gut Microbiota and Health of Zebrafish (Danio rerio). *Environ. Sci. Technol.* **2024**, *58*, 15450–15462.
